# Contextual Factors Influencing the MAMAACT Intervention: A Qualitative Study of Non-Western Immigrant Women’s Response to Potential Pregnancy Complications in Everyday Life

**DOI:** 10.3390/ijerph17031040

**Published:** 2020-02-06

**Authors:** Helle Johnsen, Ulla Christensen, Mette Juhl, Sarah Fredsted Villadsen

**Affiliations:** 1 Department of Midwifery and Therapeutic Sciences, University College Copenhagen, Sigurdsgade 26, 2200 Copenhagen N, Denmark; meju@kp.dk; 2 Section of Social Medicine, Department of Public Health, University of Copenhagen, Øster Farimagsgade 5, Postboks 2099, 1014 Copenhagen K, Denmark; ulch@sund.ku.dk (U.C.); sfv@sund.ku.dk (S.F.V.)

**Keywords:** maternal and child health, antenatal care, pregnancy complications, situational adaptation, inequity, ethnicity, migration, complex interventions

## Abstract

In western countries, immigrant women have an increased risk of negative birth outcomes. Immigrant women’s and maternity care system’s delayed response to pregnancy complications contribute to ethnic inequities in reproductive health. The MAMAACT intervention was developed to improve midwives’ and women’s response to pregnancy complications in Denmark. The study examines the context of the implementation of the MAMAACT intervention and investigates how the intended intervention mechanisms regarding response to pregnancy complications were affected by barriers in non-Western immigrant women’s everyday life situations. Twenty-one interviews with non-Western immigrant women were undertaken. Systematic text condensation and the situational-adaptation framework by Alonzo were used to analyze data. Four main categories were identified: ‘Sources of knowledge during pregnancy’, ‘Containment of pregnancy warning signs’, ‘Barriers during the onset of acute illness’ and ‘Previous situations with maternity care providers’. Attention to potential pregnancy complications may conflict with immigrant women’s everyday life situations and result in the containment of symptoms as well as causing delays in seeking medical assistance. It is probable that barriers in women’s everyday life will impact the intended intervention mechanisms and thus the full potential of the intervention may not be reached.

## 1. Introduction

Immigrants contribute to a growing part of childbirths in Denmark. Currently, approximately 20% of children are born by immigrant mothers [1]. A number of individual and organizational barriers to maternity care utilization affect immigrant women’s health [2]. A major individual barrier is a low proficiency level of the native language [2,3,4]. This barrier may be accentuated by the insufficient use of professionally trained interpreters during emergency service contacts to hospitals [5,6,7]. Challenges related to navigation within maternity care systems constitute additional barriers for non-Western migrant women [2,5].

In Europe, immigrant women’s health during pregnancy depends on conditions related to premigration, the migration process and the national setting in a new country. Studies performed within the European region have shown that non-Western immigrant women, in general, have higher risks of negative pregnancy and birth outcomes compared to the native populations [3,8,9,10]. During pregnancy, these women have an increased risk of severe maternal morbidity [10,11]. In Denmark, related tendencies are shown, as research has documented that women born in Turkey, Pakistan, and Somalia and their offspring have an increased risk of stillbirth, infant and child mortality [12,13].

Personal concepts of health and illness and health literacy are likely to impact health-seeking patterns among pregnant immigrants [14]. Compared to women coming from Western countries, immigrants from non-Western countries have been found to have lower health control beliefs [15]. Studies from England, Norway, and Sweden suggest that delays in care seeking are linked to suboptimal care and may lead to maternal mortality and stillbirth [6,7,16]. While often portrayed as an individual decision, delays in care seeking may be enforced by contextual factors beyond individual control. The World Health Organization highlights that social relations play an important role in immigrants’ health status in Western countries [3]. While social relations often are examined according to the number of personal relations, Due and colleagues argue that the impact of social relations on morbidity and mortality must be understood as structure, i.e., formal and informal social relations, as well as function such as social support, relational strain and social anchorage [17].

Currently, little is known about how immigrant women perceive and react to potential pregnancy complications. This study reports on findings part of a qualitative process evaluation [18] of the MAMAACT intervention [19]. The MAMAACT intervention was tried for effectiveness in a national realist trial from the year 2018 to 2019 [20]. Nineteen out of twenty Danish maternity wards participated in the trial. The overall aim of the trial was to reduce ethnic and social disparity in stillbirth and newborns’ health by improved management of pregnancy complications. The overall target group was all pregnant women, and the specific target group was women of non-Western origin. As a sub-study of the MAMAACT intervention, this particular study focusses on the context of the MAMAACT intervention. Moore and colleagues refer to the context of an intervention as: “*anything external to the intervention that may act as a barrier or facilitator to its implementation, or its effects*” [2,18]. Thus, understanding contextual factors is pivotal for interpreting the findings of a specific evaluation [18].

Generally, immigrant-targeted maternity care interventions are, so far, few and some initiatives lack scientific evaluation of the intervention’s implementation process as well as its effect [3]. Interventions targeting immigrant women in maternity care include the use of doulas for emotional, informational and physical support and antenatal preparation initiatives seeking to promote pregnancy and birth-related learning as well as network [21,22,23,24]. To our knowledge, initiatives that specifically focus on system and immigrant women’s response to pregnancy complications have not been examined so far [3].

### 1.1. The MAMAACT Intervention

The MAMAACT intervention was developed as a complex intervention [25] and it was tested for feasibility and acceptability from 2014 to 2015 with positive results [19]. Following this test, the intervention underwent minor adjustments and in the MAMAACT trial, the intervention comprised the following components: a six-hour training session in cultural competence and intercultural communication followed by two dialogue meetings for midwives, and a leaflet and smartphone app describing the appropriate response to warning signs during pregnancy for pregnant women. The dialogue meetings sought to boost insights from the training session and allow midwives to share their experiences of working with the MAMMACT intervention. The leaflet and app were translated into five languages (Arabic, English, Somali, Turkish and Urdu). The app was fitted with an audio function as well as a direct dial function to the local maternity ward for women with low literacy levels. The intervention was implemented universally and all women at the intervention site received the leaflet and access to the app during their first visit to the midwife. The intervention was implemented from autumn 2018 to autumn 2019.

### 1.2. Logic Model and Study Aim

**Figure 1:**
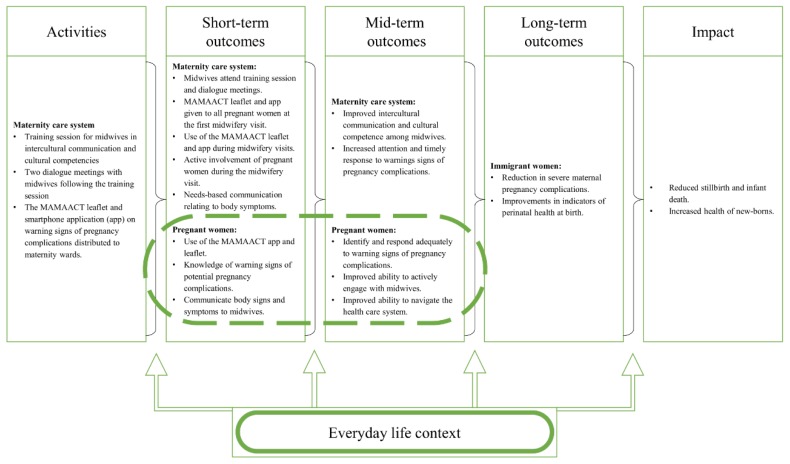
MAMAACT logic model and evaluation focus.

A logic model (Figure 1) was developed to illustrate relationships between activities, proximal and distal outcomes [18]. The evaluation drew upon principles in realist evaluation, where special attention is paid to the intervention’s circumstances [26,27]. Thus, it is anticipated that contextual factors will impact on the intended mechanisms of the intervention [18,27]. Poland and colleagues refer to the social context of the target phenomena, in this case, immigrant women’s reactions to symptoms during pregnancy and these women’s use of maternity care services [28]. Referring to Agnew [29], Poland et al. argue that to understand human behavior, it is necessary to examine micro episodes of everyday life and how these episodes are nested in specific contexts [28]. Building upon findings from the feasibility study of the MAMAACT intervention [19], we hypothesized that social life situations would affect non-Western immigrant women’s care-seeking behavior throughout the pregnancy.

The overall aim of this study was to analyze the context of the implementation of the MAMAACT intervention to investigate how the intended intervention mechanisms regarding response to pregnancy complications were affected by barriers in non-Western immigrant women’s everyday life situations.

### 1.3. Study Setting

In Denmark, antenatal care (ANC) is publicly funded and free of charge [30]. ANC is shared between general practitioners and midwives. The National Board of Health is responsible for ANC recommendations. For women with uncomplicated pregnancies, the ANC program consists of three visits to the general practitioner and five to six visits to the midwife. Women initially see their general practitioner to have the pregnancy confirmed. Following affirmation of the pregnancy, the general practitioner refers the woman to hospital-based maternity care including antenatal midwifery visits [30].

## 2. Materials and Methods

A qualitative study design [31] was chosen to illuminate non-Western migrant women’s way of adapting and reacting to their symptoms in different social situations. Due to resource restrictions, data for this study was collected at four out of ten intervention sites from autumn 2018 to summer 2019. Following recommendations from The British Medical Research Council [18], a strategic sampling approach was used to ensure that election of data collection sites included a heterogonous sample with regards to geographical location across Denmark, maternity wards serving both urban and provincial locations, variations in the number of annual births and the organization of ANC for non-Western immigrant women (Table 1).

### 2.1. Recruitment of Non-Western Immigrant Women

Midwives and an Arabic interpreter recruited 21 non-Western immigrant women for the interviews. Several women declined to participate in the interviews (number not recorded). Reasons for declining to participate were lack of time and not feeling well enough to participate in an interview. All 21 women, who consented to participate in an interview, completed the interview. Inclusion criteria were receiving the MAMAACT leaflet, being at least 28 weeks pregnant, and being at least 18 years old. As complications may develop during all pregnancies and the study by nature was exploratory, both women with uncomplicated and complicated pregnancies were invited to participate in the study. The average age among participants was 32 years. Six women were expecting their first child, eight women their second or third child, seven women their fourth child or more. The highest educational level among the women was public school (n = 17), college (n = 2), and graduate university level (n = 2). Women came from eight different non-Western countries (Iraq n = 2, Jordan n = 2, Morocco n = 2, Nepal = 1, Pakistan n = 4, Turkey n = 1, Somalia n = 3, Syria n = 5, and Yemen n = 1). Eighteen women were married or engaged and cohabiting with a male partner and three women were single and/or separated from their partner. Four of the women had experienced one or two perinatal deaths in previous pregnancies. The women had been in Denmark between less than one year and 24 years (average 7 years). All women had a Danish residence permit. Channels of immigration were family reunification (n = 11) and refugee/asylum seeker (n = 10).

### 2.2. Ethical Considerations

Women received written and verbal information about the study, and they gave written consent to participate in the study. The women’s consent form was available in Danish and Arabic. They were guaranteed personal and institutional anonymity and informed that they could withdraw from the study anytime, should they wish to do so. The study was accepted by the National Data Protection Agency (Id. No: SUND-2018-01) and The Research Ethics Committee for SCIENCE and HEALTH, University of Copenhagen (Id. No: 504-0105/19-5000).

### 2.3. Interviews with Non-Western Immigrant Women

The women chose the date, time and location of the interview. Thirteen interviews took place at an ANC facility and eight interviews took place in the women’s home. A professional interpreter assisted six interviews. A semi-structured interview guide was used to collect data [32]. During the interviews, women were encouraged to describe signs and symptoms perceived to be abnormal during their pregnancy. Women were also asked how they had managed these situations and how formal and informal social relations had influenced their adaption strategies. Spouses participated in four of the interviews. The average interview duration was one hour and five minutes. All interviews were audio-recorded and transcribed verbatim.

### 2.4. Data Analysis

Data were analyzed continuously throughout the study period to ensure sufficient information power before stopping the sampling process [33,34]. First, data were first analyzed using systematic text condensation [STC] consisting of the steps; (1) total impression; (2) identifying and sorting meaning units; (3) condensation of units and themes; (4) synthesizing [33]. Author HJ undertook analytical steps one and two. All authors discussed the remaining analytic process to ensure that the categories and sub-categories were grounded in the women’s narratives and covered the dataset as a whole. Following step four of STC, theory was applied to further focus the analysis and interpretation of study findings [33].

#### Situational Adaptation

Referring to Pawson and Tilley [26], Moore and Evans have argued that in order to understand how an intervention works in a specific context, a range of theoretical perspectives should be taken into consideration [35]. Consequently, we applied the theory by Alonzo [36] on situational-adaption to bodily signs and symptoms in order to illuminate and understand the mechanisms between the women’s everyday life context and their actual response to potential pregnancy complications.

Situational-adaption was originally developed by A.A. Alonzo to better understand social behavior surrounding disease and illness [36]. Inspired by R.J. Dubos [37], Alonzo proposes situational-adaptation to encompass health as adaption as well as the interactionist conception of the defined social situation. He uses the notion of significant others to refer to a person’s social relations. From an interactionist perspective, signs and symptoms of illness evolve in the interplay between biophysical impressions and processes of social selection, interpretation, and evaluation. These processes are often aided by informal or formal social relations. In addition, individuals who experience signs and symptoms are likely to be dependent on the situational context in which these experiences take place. Alonzo has argued that the situational-adaptation perspective is suitable for application across different disciplines. Examples of previous studies using the situational-adaptation framework include backache as an everyday illness [38], diabetes as a chronic illness [39] and cancer as a life-threatening illness [40]. Although an uncomplicated pregnancy may be viewed as a normal event in life rather than an illness [41], situational-adaptation can be useful in explaining how women perceive signs and symptoms and take action to potential complications during the pregnancy period.

Alonzo uses ‘the daily situation set to describe situations’, which are attached to a person’s social status and role set [36]. He argues that even when a person is challenged in situational accommodation, the person will maintain interactions in other situations part of everyday life. According to Alonzo, a person can exert different types of illness behavior. In this study, we draw upon two of these illness paradigms. Everyday illness refers to situations where signs and symptoms are repressed or disattended in order to manage situations. Containment is the primary adaptive process in these situations, leading individuals to keep signs and symptoms at a side level to continue daily activities and responsibilities. Acute illness behavior develops when individuals are unable to contain their signs and symptoms. Contrary to everyday illness behavior, acute illness behavior involves coping processes and the mobilization of medical resources to uphold or reestablish situational participation. Both types of illness behaviors are influenced by informal and formal social relations and the relations of power symmetry or asymmetry existing between them [36]. To add to these components, we also draw upon Gannik’s elaboration of the situational-adaptation concept [39]. She takes the perspective of disease as a social and relational phenomenon—a phenomenon developed and shaped through the interaction of people in a social everyday world. She develops a personal ‘disease model’ describing symptom control and the concept of ‘disease action fields’ as mechanisms that link structures to personal resources and action space. Thus, disease actions are framed within a life situation and are continually shaped by the action space that this total life situation and its inherent resources allow the individual [38].

In the following section, the study findings are presented. The subsequent discussion will interpret the implications of these findings for the intended mechanisms of the MAMAACT intervention.

## 3. Results

Analysis of data revealed four main categories. The main categories were ‘Sources of knowledge during pregnancy’, ‘Containment of pregnancy warning signs’, ‘Barriers during the onset of acute illness’ and ‘Previous situations with maternity care providers’.

### 3.1. Sources of Knowledge during Pregnancy

This category describes how the women navigated between different potential sources of information in situations where they needed advice.

#### 3.1.1. Use of Friends and Family for Advice

According to Gannik, advice from family and friends contributes to the development of personal disease models and hence this advice will impact how a person diagnoses and attends to bodily symptoms [38]. Findings in this study suggested that women had either limited or no access to advice from friends and family or they abstained from seeking advice from immediate family members.

These tendencies were visible in data, independent of whether women described signs and symptoms as part of everyday illness or acute illness [36], and it affected women’s possibilities for accessing and accumulating information on body symptoms in order to develop personal disease models [38].

A few of the women in this study described having relationships with female friends. Alonzo asserts that containment of signs and symptoms will be affected by ‘others’ actual assessment and response as well as the person’s perception of the evaluation of others [36]. In this study, advice from friends occurred as part of everyday interactions. Advice from friends was generally perceived to be trustworthy. Thus, advice from friends would sometimes precede and annul the necessity of seeking medical advice. This also seemed to be the case when this advice conflicted with advice from maternity care providers. For example, one woman, who was being followed for fetal growth retardation, had told some friends that she was experiencing a reduction in fetal movements. Her friends had responded by advising her how to respond to the situation:

“… *I told them, that I hadn’t felt the baby kick, I felt nothing. They recommended eating something sweet because it would get the baby moving again*”.

(Pregnant woman, Syria, I21)

The woman’s friends had given her a chocolate sweet. After eating the chocolate, the woman had felt the baby move again and as a result, she had found eating chocolate to be an appropriate response to symptoms related to lack of fetal movements.

Although some women talked to their mother or sister about their pregnancy, these conversations were described as more superficial, for example, conversations on the baby’s gender, the growth of the baby or how far along the pregnancy was. Several women in this study had experienced complications during their pregnancy. However, most of them had not informed their family of these situations. Some women described having families in countries with poor internet coverage. Thus, contact with family members would be irregular and not necessarily available when needed. In addition, some women had parents and siblings in countries hampered by war and poverty. Keeping information about problems during pregnancy from the family served to protect them from concerns and further stress:

“…. *you don’t talk about the pregnancy, they (my parents) have plenty they must to deal with*”.

(Pregnant woman, Iraq, I18)

Some women had no relatives in Denmark. Other women had the family of the spouse as the nearest relatives. Although the mother-in-law was described to be a dominant family figure, a number of women in this study were hesitant to use the spouse’s mother-in-law or sisters as an aid for pregnancy-related advice. This was also the case, when women lived together with the spouse’s family or if his family lived nearby. Several barriers kept women from seeking their support. Some women felt they did not know the family of the spouse well enough to seek their advice. Others were concerned about issues of confidentiality as the pregnancy was considered an intimate and private condition:

“… *I don’t have any close female relations or family members here. I have the two sisters of my spouse and they are a little older than me and have both given birth, but I don’t ask them (about my pregnancy). This is not something you talk about*”.

(Pregnant woman, Pakistan, I19)

Gannik argues that it takes time to build personal disease models [38]. Thus, family advice is an important contributor to enabling people to plan their disease actions. She refers to tight and loose family situations as situations with varying degrees of freedom [38]. Lack of actual access to women’s own family and hesitance to use the spouse’s family for pregnancy-related advice in this study, can therefore be seen as tight situations affecting the women’s conditions for building their diagnose of and reaction to symptoms on experiences and recommendations from their family [38].

#### 3.1.2. Dealing with Private Matters Online

According to Gannik, people are likely to draw upon information from different types of media when restructuring knowledge into their personal models of disease [38]. She explains that people will integrate media information with information from family, friends and health care professionals. In this study, women used the internet to compensate for the lack of advice from their family and friends. Several advantages were associated with the use of the internet. Women were able to access immediate information regarding their symptoms and thus this strategy enabled them to manage symptoms without having to travel for medical advice. The main motivation for using the internet as an information source was that women were able to seek information anonymously, in situations where symptoms were seen as being of a more private nature. Some women would enter chat fora and use other women’s experiences of pregnancy complications as a source of advice. However, entering personal pregnancy symptoms in these fora was considered inappropriate. Using hospital websites for information was rare among the women. The main strategy for retrieving information was ‘googling’. Women would enter their symptoms and then use the immediately suggested links. Online information was generally considered to be reliable and thus women would not investigate who owned the particular website. One woman described how she, during the second trimester of her pregnancy, had experienced vaginal discharge of watery fluid. She had been concerned that these symptoms indicated a premature rupture of the membranes. Although she knew that the symptom could potentially be a sign of acute illness [36] she adapted by seeking advice online instead of seeking medical assistance:

“… *When I googled (my symptoms) … I was confirmed in my suspicion that I have in fact overexerted myself and I have been too tired…Plus the pressure from the baby also leads to fluid from the lower regions*”.

(Pregnant woman, Jordan, I7)

The woman did not discuss the incident with her doctor or her midwife as she felt the information she found on the internet was sufficient to diagnose her symptoms and thus further actions were not needed [38].

### 3.2. Containment of Pregnancy Warning Signs

This category illuminates how women adapted to situations where they experienced signs and symptoms of potential pregnancy complications and how their daily situation set impacted these situations.

#### Suppressing Symptoms and Postponing Medical Attention

Alonzo refers to everyday illness as signs and symptoms which can be contained by levels of side involvement so that they may be integrated into activities, role demands, and obligations of the situation [36]. Women in this study described having many domestic responsibilities resulting in limited action space [38] for self-care. As they depended on their spouse to provide economically for the family, the spouse would spend many hours working away from home. Providing care for the rest of the family included tasks such as delivering and picking up children from daycare, shopping groceries, cooking, cleaning and doing laundry. According to Alonzo, commitment may be driven by perceptions of identity and attachment. He also claims that commitment to situations will influence a persons’ attention to and the meaningfulness of signs and symptoms [36]. Several women described symptoms such as headaches, stomachaches, backaches, and contractions of the uterus. As most of the women lacked a social network to help them with household responsibilities, they would repress symptoms for the home to function as intended. As bodily expectancies are likely to influence the assessment of signs and symptoms [36], some women tended to explain their symptoms as body reactions to the strain of domestic work, as exemplified by the following narrative:

“*I am always in pain. I can’t always cope… I get exhausted because there is too much work*”.

(Pregnant woman, Somalia, I3)

This woman did not attend the different body pains she experienced as she concluded they were a result of strain caused by numerous tasks within the home setting.

Women who worked or went to school also carried many domestic responsibilities. Gannik uses the term ‘the disease action field’ to reflect the freedom to act within the present life situation [39]. This includes structural factors such as education, work, and income that affect both economic and time resources. Women in this study were unaware of their rights when experiencing potential pregnancy warning signs. As they were highly dependent on their income or social benefits to provide for the family, they feared possible economic consequences of seeking medical assistance during work or school hours. Perceived or actual lack of flexibility in these women’s conditions was illustrated by a high sense of duty directed towards maintaining these obligations despite experiencing pregnancy warning signs:

“…*My spouse told me, you can’t go to work, you were ill yesterday. I tell him no! I have to go to work because two of my colleagues were off work ill…It won’t work if I stay home… so I went to work…when I came home it was really bad, a lot of pain in my stomach…I had pain for four to five minutes at a time. I have never tried that before, I got scared, will I deliver now, before my due date?*”.

(Pregnant woman, Morocco, I20)

Instead of using emergency maternity services, some women would wait to present their symptoms until they had a scheduled appointment with either their general practitioner or their midwife resulting in delays in medical attention to symptoms. Among reasons for this tendency was that scheduled appointments fitted better with women’s work obligations (I20) and that women felt personal doctors and midwives knew the women’s obstetric history better than maternity care providers at the hospital and thus were more qualified to assess their symptoms (I12, I21). One woman described how she was concerned about intermittent stomach pain during pregnancy. She depended on her husband to help her explain these symptoms to the midwife. As the woman’s husband needed to plan time off work, she would use her scheduled appointment to discuss her symptoms:

“…*it (my midwifery appointments) depend on what is feasible for my husband…I get to ask about things (symptoms) I don’t understand. I like when he is present*…”.

(Pregnant woman Syria, I21)

### 3.3. Barriers during the Onset of Acute Illness

This category illustrates how a lack of informal social relations and obligations to existing children impacted women’s possibility to seek medical assistance.

#### 3.3.1. Someone to Advocate for Medical Assistance Needs

Alonzo uses the term ‘type lV situations’ to illustrate situations where a person must shift from containment strategies excluding medical assistance to an implied use of resources such as medication and health care professionals [36]. However, in these situations, some of the women depended on others to facilitate this medical assistance. Spouses who knew the Danish language were reported as having a key role during these situations as they were able to call maternity emergency services. If the couple owned a vehicle, the spouse would also provide transport to the local maternity ward. When women were unable to reach their spouse during emergency situations or they did not have a spouse, they instead depended on immediate help from other informal social relations, such as neighbors. Some women found it inappropriate to ask their neighbors for help, as they did not engage with them on a daily basis. A few women had received help from their neighbors. For example, one woman who did not speak Danish had her neighbor call the local maternity ward when she was bleeding (I13). Another woman had a neighbor help her call for an ambulance (I20). Nevertheless, the general tendency among the women was that neighbors were not part of their social relations and thus the women felt more vulnerable during pregnancy and birth. One woman described how moving out of the asylum center had changed her situation significantly:

“… *I am worried. At the asylum center, many people could help, many people living together…women who could help, who had children, so they knew. They helped me a lot because eight families lived in every building. There were also people working in the office who could step in and help me, call for an ambulance…But now no one*”.

(Pregnant woman, Syria, I16)

#### 3.3.2. Looking After Existing Children

Acute illness behavior implies a shift in situational attention [36]. In recognition of being unable to contain symptoms over a significant segment of situations, the person is forced to seek medical attention [36]. Yet, to do so, role relationships with others will need to be attenuated. Alonzo asserts that although acute illness is the main focus, everyday concerns, obligations, and commitments, for example, related to the immediate family, may be difficult to set aside [36]. In this study, existing children were described to be a major barrier for seeking immediate medical attention when experiencing signs and symptoms of acute illness during pregnancy. One woman described how she and her spouse had needed to go to the hospital during the middle of the night. As they had no one to step in, they had left their children sleeping at the house (I12). Delaying departure to the hospital during birth was a considerable concern among the women. A woman described how the only support she had in acute illness situations was the spouse’s sister who had between one and two hours of transport to their home (I16). Another woman described how she had recently moved to a new house with her children and thus did not know any of her neighbors (I5). She had a boyfriend and a friend who could help. However, they did not live nearby. The woman had previously delivered unexpectedly at home late at night. To prepare herself for a similar situation, she had watched videos on Youtube so she knew how to take action in a similar situation:

“…*What am I going to do, tell my children ‘wake up, help me!’. They will be asleep and they won’t hear me…I will need my telephone…will need to sit on the floor, so the baby won’t fall out…you take the baby out this way…they (the videos) showed me what to do if you are all alone*”.

(Pregnant woman, Yemen, I5)

### 3.4. Previous Situations with Maternity Care Providers

This category portrays how experiences of previous and recent encounters with maternity care providers impacted women’s adaptation strategies in acute illness situations.

#### 3.4.1. Concerns of Maternity Care Providers Reactions

Seeking medical assistance is, according to Alonzo, considered to be the primary adaptation strategy in situations where a person experiences signs and symptoms of acute illness [36]. However, the decision to seek medical assistance may according to Gannik be influenced by personal experiences and contacts with health care professionals [38]. In this study, perceptions of maternity care providers had a great influence on women’s adaption strategies when experiencing potential pregnancy complications. While some women had positive experiences of previous situations with maternity care services, other women had more negative experiences. Perceptions of not being taken seriously or being listened to when presenting symptoms were described to lead to negative experiences. For example, one woman described how her water had broken at the hospital preterm, but the doctor had explained the fluid as urine (I5). She had told the doctor that she had delivered before and that she knew how amnion fluid looked. Another woman described how she, during her previous pregnancy, had an acute caesarian section because the doctor and midwife thought she was able to have a natural birth:

“*I told my doctor before and also the midwife during the pregnancy, ‘I cannot birth normally’…when I was in Syria…she (the doctor) told me ‘you cannot birth normally’…there was not enough room in my pelvis…It makes me absolutely angry…the (Danish) doctor’s job is to listen to me*…”.

(Pregnant woman, Syria, I17)

Enduring long periods of time at the maternity ward without being attended to and being sent home also affected women’s motivation to contact maternity emergency services:

“…*Three hours I am sitting freezing in the waiting room…no one comes and tells me I can lie down or brings me a blanket, nothing! I would rather avoid having to engage with them and ask them (maternity care providers)…it’s neglect of care, no one asks me if I would like to stay the night at the hospital…I am pregnant, I have no one, no network, someone beside me to take care of me*”.

(Pregnant woman Syria, I13)

#### 3.4.2. Preferring Self-Care

To avoid potentially stressful encounters with maternity care providers, some women turned to self-care practices instead. Alonzo claims that situational resources are needed if signs and symptoms are to be managed [36]. Situational resources include actions involving others or things perceived to benefit both the management of signs and symptoms as well as the adaptation to the specific situation. Women in this study drew upon a number of resources to contain their situation outside the maternity care setting. According to Alonzo, resources may be used for purposes of immediate adaptation [36]. For example, one woman described how she had taken to bedrest until she felt better after passing out (I13). This strategy had been successful and thus she did not feel a need to contact maternity emergency services. Other women would use resources for the purpose of sustained adaptation, that is actions perceived to result in adaptation over longer periods of time [36]. This strategy was seen in the description from a woman, who had been concerned about fetal movements during her pregnancy following a previous stillbirth. To calm herself she had bought a fetal heart monitor device. Although she had the device, she had also contacted the local maternity ward on several occasions due to her concerns. The woman had contacted the ward when she was 24 weeks pregnant. She was not feeling enough fetal movements and had assessed that she needed an ultrasound examination to examine the baby’s wellbeing. Upon experiencing the maternity care providers’ response to her situation, she decided to deal with the situation on her own instead:

“… *In week 24 I call the ward and say… ’the baby is not very active’. They tell me ‘you know this is normal at this time of the pregnancy’. I ask them ‘could you just examine me for a few minutes? I can come in’…They tell me ‘no, if you come in, we are only able to listen to the heartbeat, and if it’s fine, that’s it’. I tell them ‘okay then I won’t bother. I have a device’. I am able (to listen to the heartbeat)*”.

(Pregnant woman, Pakistan, I12)

## 4. Discussion

In the following discussion findings related to informal and formal social relations, containment of signs and symptoms and potential delays in care will be further interpreted against the intended mechanisms of the MAMAACT intervention.

### 4.1. Having Informal Social Relations

According to both Alonzo and Gannik, informal social relations play a key role in how a person adapts to signs and symptoms [36,38]. A recent synthesis of qualitative literature by Merry and colleagues found that during parenthood immigrant’s experiences are impacted by a continuing and pervasive transnational dimension [42]. This was due to immigrants maintaining ties with their home country in their new country of residence. The synthesis found that immigrants relied on their families for social support and advice. Similarly, the Danish National Board of Health has stated that during pregnancy, family structures and especially the mother-in-law plays an important role in immigrant women’s perceptions of health, pregnancy, and birth [30]. According to Uchino and colleagues as well as Due and colleagues, social support and the functions of social relations are key for the prevention of morbidity and mortality [17,43]. Among dimensions of the function of social relations in the model developed by Due et al. is social support, including emotional support and informational support [17]. While some women in this study drew upon family members for emotional support, the protection of parents in the home country and taboos surrounding the pregnancy condition kept women from using family members for informational support. These findings highlight the relevance of the MAMAACT leaflet and app as this material provided women with information as well as graphic illustrations on which symptoms of potential pregnancy complications women should respond to. The material also provided contact information on the local emergency maternity care services as well as an auto-dial function in the app. However, findings in this study showed that some women would draw upon the internet or friends for information instead of contacting maternity care providers as specified in the MAMAACT material. Thus, having the MAMAACT material was not sufficient for all the women to respond to pregnancy complications as intended. Findings also showed that women found informal social relations and social media to be trustworthy, suggesting that these women may not have had the necessary health literacy skills [44] to appraise information provided by informal social relations or social media. In addition, Kleinman and Benson have argued that cultural differences in health-related beliefs and behaviors affect the interactions between users and health care systems [45]. Thus, another reason for women’s high use of social media could be that social media provided information from their country of origin, which fitted the women’s cultural health beliefs regarding their pregnancy situation.

Due and colleagues use the term ‘instrumental support’ to describe the provision of practical help in everyday life [17]. Several women in this study suffered from social isolation. Thus, Danish maternity care wards may overestimate and over-rely on family support among immigrant women. International studies have previously documented similar findings showing that immigrant women may be socially isolated in their new country of residence and that lack of social support may prevent immigrant women’s use of maternity care services [2,3,5]. In this study, some women described lacking practical help in situations where emergency medical assistance was needed. These tendencies were especially dominant in situations where women had more than one child. The MAMAACT intervention sought to increase immigrant women’s use of emergency maternity care services when experiencing potential pregnancy complications. However, for some women, social isolation could function as a barrier.

### 4.2. Containment of Signs and Symptoms in Everyday Situations

According to Alonzo, situational commitment is related to a person’s identity [36]. In a previous study by Hansen and colleagues which investigated the management of type 1 diabetes in the context of work, the authors found that participants experienced tensions between logics of being a patient and logics of being a worker [39]. Similarly, women in this study navigated between several identities such as being pregnant, being the main care provider and being a student or an employee. Having to navigate between several identities and the obligations associated with each identity affected women’s adaptation strategies.

Alonzo uses the term ‘drifting’ to describe how a person drifts between health and illness or adaptation and non-adaptation [36]. Findings in this study suggested that some women would explain signs and symptoms of potential pregnancy complications as everyday illness situations or that they would contain them to meet obligations. In some cases, women did not notify maternity care providers of their experiences, in other cases women would postpone medical attention until a coming scheduled appointment. An important aspect of the MAMAACT intervention was to promote timely response to pregnancy complications among immigrant women. Hence, these adaptation strategies conflicted with the components of the MAMAACT intervention and point towards the need for maternity care systems to understand how the complexity of everyday life situations may lead to postponing medical attention.

### 4.3. The Importance of Relations with Maternity Care Providers

Alonzo asserts that seeking medical consultation differentiates acute illness from everyday illness [36]. Poor experiences from previous encounters with maternity care providers had a great impact on women’s motivation to seek medical assistance in this study. Several studies have highlighted the importance of immigrant’s experiences with health care services for their health-seeking practices. Previous studies point to experiences of poor encounters with maternity care providers to be caused by perceptions of being stereotyped and discriminated due to race [46,47]. In addition, studies point to trust in maternity care services and maternity care providers as impacting immigrant women’s attendance to maternity services as well as their compliance with maternity care providers’ recommendations [7,16]. Findings in this study showed that women took to what Alonzo calls self-imposed care [36]. This highlights the importance of establishing trust-based relationships with women in addition to training midwives in intercultural communication and cultural competence. As shown in this study, women were highly influenced by their encounters with maternity care providers during their previous as well as their present pregnancies. Furthermore, self-imposed care added to these women’s vulnerability as many of them lacked informal social relations to support them and thus relied more on maternity care providers as formal social relations [17] for support. Hence, further efforts are needed to increase immigrant women’s trust in maternity care providers and maternity care systems. Studies outside the field of maternity care point to the importance of professionally trained interpreters knowledgeable of the particular language as well as the cultural practices in the area of origin to enhance alliances between care providers and immigrant patients [48]. These studies also point to the importance of continuity of interpreters in care in order to build trusting relationships between the patients and care providers but also between the patients and interpreters [48].

### 4.4. Multiple Routes to a Delayed Response to Pregnancy Complications

Findings in this study showed that containment of signs or symptoms of potential pregnancy complications resulted in a lack of or delayed response to potential pregnancy complications among some of the women. The three delay framework has previously been used to investigate immigrant women’s decision to seek care when suspecting obstetric problems [7,16]. These studies found that delays in immigrant women’s decisions to seek care were mainly caused by a lack of trust in maternity care systems and low language proficiency [7,16]. Although similar findings are presented in this study, the analysis of data also revealed several additional conditions impacting women’s decision to seek care, such as lack of informational support, issues regarding the appraisal of lay information sources, lack of practical help, and dependence on employment to provide for the family. These findings show how limited action space in immigrant women’s everyday life had a profound impact on their opportunity to build personal disease models and take disease actions [38]. Veenstra and Abel have argued that more attention should be directed towards the dynamics between economic capital such as income, cultural capital such as formal education and social capital such as the network of social ties in the production of health inequalities [49]. The authors refer to the capital multiplier interplay where the application of one form of capital toward producing good health is facilitated by the possession of another form of capital. In this study, the analysis of data revealed multiple types of capital interplay. For example, lack of economical capital and lack of social capital negatively impacted women’s possibility to seek medical assistance.

Although the MAMAACT intervention could not attend broader socioeconomic inequalities among immigrant women, it did address several barriers identified in this study, suggesting the relevance of the intervention despite these barriers. The material provided reliable information on pregnancy complications as well as contact information to women who were liable to lack informational support from social relations. For women with low Danish language proficiency, this information was available in the most dominant non-Western languages. In addition, the training of midwives in intercultural communication and cultural competence sought to improve midwives’ sensitivity to immigrant women’s individual needs. Study findings also indicate that additional initiatives are needed to support immigrant women’s response to pregnancy complications. Such initiatives should focus on network building and peer support among immigrant women, but also on maternity care system flexibility to improve immigrant women’s access to care and their trust in maternity care services. These initiatives could include the establishment of outreach clinics and domiciliary-based maternity care.

### 4.5. Strengths and Limitations

Although women included for this study only represented four of the intervention sites, these intervention sites were diverse according to their geographical location, size, and organization of ANC. Midwives and an interpreter recruited women for the study, which can have resulted in the preference of women with certain characteristics. These sampling procedures may have led to selection bias [31] and they affect the interpretation of data findings. In the sample of women, 18 out of 22 women cohabited with a male partner. In Denmark, it is estimated that one out of three mothers of non-Western origin is single [1]. In addition, single mothers with non-Western origin have more children than mothers of Danish origin [1]. This lack of heterogeneity may affect the generalization of study findings [31]. Furthermore, spouses participated in four of the interviews, which may have restricted these women’s opportunities to speak freely during the interview. However, we consider the heterogeneity in the sample regarding women’s country of origin a strength in this study. Also, to the authors’ knowledge, this is the first time that the situational-adaptation framework has been applied to the reproductive health field. The use of this framework increases the transferability of findings to other settings within and outside the field of maternity care [31].

## 5. Conclusions

This study contributes with novel in-depth knowledge of immigrant women’s adaptation strategies and the importance of functional social relations for the response to pregnancy complications. The findings show that rather than understanding signs and symptoms of potential pregnancy complications as isolated events these situations may conflict with immigrant women’s everyday situation set and thus result in the containment of signs and symptoms as well as causing delays in seeking medical assistance. Numerous factors prevented the women from seeking timely care, including conflicting lay advice, lack of informal social relations for information support and practical help as well as poor experiences with maternity care providers. The MAMAACT intervention addressed several of the barriers identified in the study. However, it is also probable that these barriers will impact the intended mechanisms of the intervention in such a way that the potential of the intervention might not be fully reached. Broader socioeconomic inequalities are likely to reproduce ethnic disparities in reproductive health, and additional maternity care initiatives are needed to address immigrant women’s distinct needs.

## Figures and Tables

**Table 1:** Data collection sites.

Maternity Ward	Annual Births Above or Below 3000	Location in Denmark East/West	Populations Served Urban/Provincial	Immigrant Targeted Antenatal Care
1	Above	East	Urban	No
2	Below	East	Provincial	Yes
3	Above	West	Urban	Yes
4	Below	West	Provincial	No
